# Downregulation of *COL12A1* and *COL13A1* by a selective EP2 receptor agonist, omidenepag, in human trabecular meshwork cells

**DOI:** 10.1371/journal.pone.0280331

**Published:** 2023-01-11

**Authors:** Masashi Kumon, Masahiro Fuwa, Atsushi Shimazaki, Noriko Odani-Kawabata, Ryo Iwamura, Kenji Yoneda, Masatomo Kato

**Affiliations:** 1 Product Development Division, Santen Pharmaceutical Co., Ltd., Nara, Japan; 2 Product Development Division, Santen Pharmaceutical Co., Ltd., Osaka, Japan; 3 Pharmaceutical Division, Pharmaceuticals Research Laboratory, UBE Corporation, Yamaguchi, Japan; Oregon Health and Science University, UNITED STATES

## Abstract

Omidenepag isopropyl (OMDI) is an intraocular pressure (IOP)-lowering drug used to treat glaucoma. The active form of OMDI, omidenepag (OMD), lowers elevated IOP, the main risk factor for glaucoma, by increasing the aqueous humor outflow; however, a detailed understanding of this mechanism is lacking. To clarify the IOP-lowering mechanism of OMDI, the effects of OMD on the mRNA expression of the extracellular matrix, matrix metalloproteinases (MMPs), and tissue inhibitors of metalloproteinases (TIMPs) were evaluated in human trabecular meshwork cells. Under 2D culture conditions, the mRNA expression of *FN1*, *COL1A1*, *COL1A2*, *COL12A1*, and *COL13A1* decreased in a concentration-dependent manner after 6 or 24 h treatment with 10 nM, 100 nM, and 1 μM OMD, while that of *COL18A1* decreased after 6 h treatment with 1 μM OMD. Significant changes in expression were observed for many MMP and TIMP genes. Under 3D culture conditions, the extracellular matrix-related genes *COL12A1* and *COL13A1* were downregulated by OMD treatment at all three concentrations. Under both 2D and 3D culture conditions, *COL12A1* and *COL13A1* were downregulated following OMD treatment. Reduction in the extracellular matrix contributes to the decrease in outflow resistance, suggesting that the downregulation of the two related genes may be one of the factors influencing the IOP-lowering effect of OMDI. Our findings provide insights for the use of OMDI in clinical practice.

## Introduction

Glaucoma, the leading cause of irreversible blindness, is an ocular neurodegenerative disease characterized by selective loss of retinal ganglion cells resulting in progressive visual field defects [1–4]. The only evidence-based treatment for glaucoma is intraocular pressure (IOP) reduction [5–7]. Prostaglandin F (FP) receptor agonists are mainly used as first-line treatment agents for glaucoma [8,9]; however, they are occasionally associated with the topical ocular side effect of prostaglandin-associated periorbitopathy (PAP), which may limit their clinical use [10–12]. Therefore, alternative treatment options with better safety profiles and novel mechanisms of action are needed.

Omidenepag isopropyl (OMDI) is the first prostaglandin E_2_ receptor EP2 subtype (EP2 receptor) agonist used as an IOP-lowering drug for glaucoma treatment worldwide, approved in Japan in 2018, followed by Korea and other Asian countries [13,14]. OMDI exerts potent IOP-lowering effects in both animals [15,16] and humans [17–19]. OMDI ophthalmic solution of 0.002% has been used for the treatment of glaucoma and ocular hypertension [13,14]. OMDI is a prodrug that improves the corneal permeability of omidenepag (OMD), the active form of OMDI, which has a novel non-prostaglandin chemical structure that differs from that of any existing prostaglandin analog, including FP receptor agonists, and highly selective EP2 receptor agonist activity [20]. A multicenter, double-blinded, controlled phase 3 clinical study (AYAME Study [NCT02623738]) [18] demonstrated that OMDI is safe and tolerable and has noninferior IOP-lowering efficacy comparable to that of latanoprost, the FP receptor agonist. In addition to monotherapy, we previously demonstrated that OMDI exerted additive IOP-lowering effects when combined with timolol (β-adrenergic antagonist), brinzolamide (carbonic anhydrase inhibitor), netarsudil and ripasudil (Rho-associated coiled-coil containing protein kinase inhibitor), or brimonidine (α_2_-adrenergic agonist), in conscious ocular normotensive monkeys [16]. Furthermore, unlike FP receptor agonists, previous nonclinical studies have suggested that OMDI does not cause PAP [21,22]. Considering its mechanism of action, which involves stimulating the EP2 receptor followed by increasing the aqueous humor outflow through both conventional and uveoscleral outflow pathways [23], IOP-lowering efficacy, and safety, OMDI is a potential alternative first-line therapeutic agent to FP receptor agonists.

IOP is regulated by resistance to aqueous humor outflow caused by the extracellular matrix (ECM) within the deepest portion of the trabecular meshwork (TM) and basement lamina of the Schlemm’s canal (SC) inner wall endothelium [24–26]. Elevated IOP is the only validated risk factor for glaucoma [6,27]; hence, IOP reduction remains the primary goal of therapy [5,7]. The main reason for the elevated IOP in primary open-angle glaucoma, the major subtype of glaucoma, is increased resistance in the conventional outflow pathway consisting of the TM and SC [24]. We previously demonstrated that OMDI lowers IOP by increasing the aqueous humor outflow through both the conventional and uveoscleral outflow pathways in ocular hypertensive monkeys [23].

This mechanism of action is unique to OMDI because the FP receptor agonists and the other EP2 receptor agonist butaprost with prostaglandin structure and broad affinity to other prostanoid receptors predominantly stimulate the uveoscleral outflow pathway [28,29]. Although OMD can modulate TM cell fibrosis and SC endothelial cell permeability [30], a detailed understanding of the molecular mechanism of action of OMDI facilitating aqueous humor outflow is lacking. Thus, to clarify the IOP-lowering mechanism of OMDI, especially focusing on the conventional outflow pathway, we evaluated the effects of OMD on the mRNA expression of the ECM, matrix metalloproteinases (MMPs), and tissue inhibitors of metalloproteinases (TIMPs) associated with aqueous humor outflow resistance [24–26] using quantitative real-time PCR in two- and three-dimensionally (2D and 3D) cultured human TM (HTM) cells. Clarification of the mechanism of OMDI in the conventional outflow pathway provides reference information for determining the order of drug use initiation combined with existing anti-glaucoma drugs, which will lead to more effective use in clinical practice.

## Materials and methods

### Materials

OMD was synthesized by UBE Corporation (Yamaguchi, Japan) and dissolved in dimethyl sulfoxide (DMSO [Nacalai Tesque, Kyoto, Japan]).

### Culture of primary human trabecular meshwork (HTM) cells

Primary HTM cells were obtained from ScienCell Research Laboratories (Carlsbad, CA, USA). HTM cells were isolated from a single donor. To characterize these HTM cells, we confirmed myocilin (MYOC) upregulation induced by dexamethasone (DEX) treatment according to the procedure described in previous reports [31–34] (S1 Fig). HTM cells were maintained in the supplemented manufacturer-specified medium, namely, trabecular meshwork cell medium (TMCM) containing 2% fetal bovine serum, the manufacturer-specified supplement (i.e., undisclosed growth factors, hormones, and proteins), 100 units/mL of penicillin, and 100 μg/mL of streptomycin (ScienCell Research Laboratories). HTM cells were cultured in a poly-L-lysine-coated flask, and the supplemented TMCM was exchanged every 3–4 d. HTM cells were used between passages 3 and 5 in the present study.

### *In vitro* cell viability assay

HTM cells were seeded in 96-well plates pre-coated with poly-L-lysine (AGC Techno Glass Co., Ltd., Shizuoka, Japan) at a density of 1.0 × 10^4^ cells/well. The cells were placed in a 5% CO_2_ incubator at 37°C and cultured in supplemented TMCM. The HTM cells reached confluence at 24 h after seeding, following which, the supplemented TMCM was removed and the cells were washed once with non-supplemented TMCM. The cells were incubated for 24 h with OMD at 10 nM, 100 nM, and 1 μM in non-supplemented TMCM containing 0.1% DMSO, while non-supplemented TMCM containing 0.1% DMSO was used as the vehicle, and the cell viability was assessed using 3-(4,5-dimethylthiazol-2-yl)-5-(3-carboxymethoxyphenyl)-2-(4-sulfophenyl)-2H-tetrazolium (MTS) assays in accordance with the manufacturer’s instructions (CellTiter 96 AQ_ueous_ One Solution Cell Proliferation Assay, Promega, Madison, WI, USA).

### Drug treatment of 2D- and 3D-cultured HTM cells

For the 2D culture, HTM cells were seeded in supplemented TMCM in 12-well plates pre-coated with poly-L-lysine (AGC Techno Glass Co., Ltd., Shizuoka, Japan) at a density of 1.2 × 10^5^ cells/well. The HTM cells reached confluence at 24 h after seeding. Subsequently, the supplemented TMCM was removed and the confluent cells were washed once with non-supplemented TMCM. The cells were treated with OMD at 10 nM, 100 nM, and 1 μM in non-supplemented TMCM containing 0.1% DMSO for 6 or 24 h, while non-supplemented TMCM, containing 0.1% DMSO was used as the vehicle.

For the 3D tissue generation, a 3D culture of HTM cells was performed according to a previous study [35], with modifications. Briefly, HTM cells were suspended in Tris-buffered saline containing 150 mM sodium chloride and 0.04 mg/mL gelatin (Wako Pure Chemicals, Osaka, Japan) with gentle shaking for 30 min at approximately 20–25°C. The cells were briefly centrifuged at 500–600 g and resuspended in supplemented TMCM. The cells were then seeded in 24-well plate cell culture inserts (0.4-μm transparent PET membrane; Corning, Corning, NY, USA) coated with 0.12 mg/mL fibronectin (Sigma-Aldrich, Saint Louis, MO, USA) at a density of 5.0 × 10^5^ cells/well. To generate stable 3D-cultured tissues that exhibit strong three-dimensional cell–cell adhesion, the HTM cells were cultured in supplemented TMCM for 6 d. Subsequently, the supplemented TMCM was removed, and the cells were washed once with non-supplemented TMCM. The cells were treated with OMD at 10 nM, 100 nM, and 1 μM in non-supplemented TMCM containing 0.1% DMSO, for 6 or 24 h, while non-supplemented TMCM containing 0.1% DMSO was used as the vehicle.

### RNA extraction, reverse transcription, and quantitative real-time PCR

After 6 and 24 h of OMD treatment, total RNA was extracted from the 2D- and 3D-cultured HTM cells using RNeasy Plus Universal Mini Kit (Qiagen, Hilden, Germany) in accordance with the manufacturer’s instructions. For the reverse transcription and quantitative real-time PCR, the experiment was performed as previously described [36,37], with minor modifications. Briefly, total RNA was used for reverse transcription to synthesize complementary DNA (cDNA) by means of QuantiTect Reverse Transcription kit (Qiagen) in accordance with the manufacturer’s instructions. Real-time PCR was performed using either the Mx3005P Real-Time PCR System (Agilent Technologies, Santa Clara, CA, USA) with QuantiFast SYBR Green PCR Kit (Qiagen) or the QuantStudio 3 Real-Time PCR System (Thermo Fisher Scientific) with PowerUp SYBR Green Master Mix (Thermo Fisher Scientific) in accordance with the manufacturers’ instructions. The expression of each gene was determined by the standard ΔΔCt method. The relative expression level of target genes (*FN1*, *COL1A1*, *COL1A2*, *COL4A2*, *COL12A1*, *COL13A1*, *COL18A1*, *MMP1*, *MMP2*, *MMP3*, *MMP9*, *MMP11*, *MMP12*, *MMP14*, *MMP15*, *MMP16*, *MMP17*, *MMP24*, *TIMP1*, *TIMP2*, *TIMP3*, and *TIMP4*) was normalized to that of *GAPDH* and presented as percentages relative to the vehicle-treated group. Some of the pre-designed primers were purchased from Takara Bio (Shiga, Japan). The sequences of the primers or part numbers of the pre-designed primers for the evaluated genes are shown in Table 1.

**Table 1:** Primers used for quantitative real-time PCR.

Gene	Primer set P/N	Forward (5′–3′)	Reverse (5′–3′)
*GAPDH*	HA067812	-	-
*FN1*	-	AGCGGACCTACCTAGGCAAT	GGTTTGCGATGGTACAGCTT
*COL1A1*	-	GAGAGCATGACCGATGGATT	CCTTCTTGAGGTTGCCAGTC
*COL1A2*	-	GAGGGCAACAGCAGGTTCACTTA	TCAGCACCACCGATGTCCAA
*COL4A2*	-	CCACAGTCAGGATGTCTCCATC	CGGTGACACCAGTGATTGGC
*COL12A1*	-	CCACAGGTTCAAGAGGTCCC	TGTGTTAGCCGGAACCTGGA
*COL13A1*	HA144155	-	-
*COL18A1*	-	GGCACGCATCTTCTCCTTT	CACGATGTAGGCGTGATGG
*MMP1*	HA169158	-	-
*MMP2*	HA173965	-	-
*MMP3*	HA160220	-	-
*MMP9*	HA129244	-	-
*MMP11*	HA154311	-	-
*MMP12*	HA122565	-	-
*MMP14*	HA274826	-	-
*MMP15*	HA125534	-	-
*MMP16*	HA250859	-	-
*MMP17*	HA153284	-	-
*MMP24*	HA269320	-	-
*TIMP1*	HA257212	-	-
*TIMP2*	HA143999	-	-
*TIMP3*	HA269773	-	-
*TIMP4*	HA157583	-	-

Primer sets with P/N were pre-designed and purchased from Takara Bio (Shiga, Japan). Other primers’ sequences are shown.

### Statistical analysis

Each value depicted in the figures represents the mean ± S.E. All statistical analyses were performed using EXSUS software version 10.0.7 (EP Croit, Tokyo, Japan) in accordance with the manufacturer’s instructions. For *in vitro* cell viability assay, Bartlett’s test for equal variance was performed followed by Dunnett’s test for multiple comparisons in parametric method. For quantitative real-time PCR analysis, after Bartlett’s test for equal variance, Dunnett type multiple comparisons were performed in the following manner: (a) If the data were non-equal variance (P < 0.05), the data were calculated using the parametric method following Log transformation; (b) In the case of equal variance (P ≥ 0.05), the parametric method was used. Differences were considered significant at P < 0.05.

## Results

### Effect of OMD on HTM cell viability

**Fig 1:**
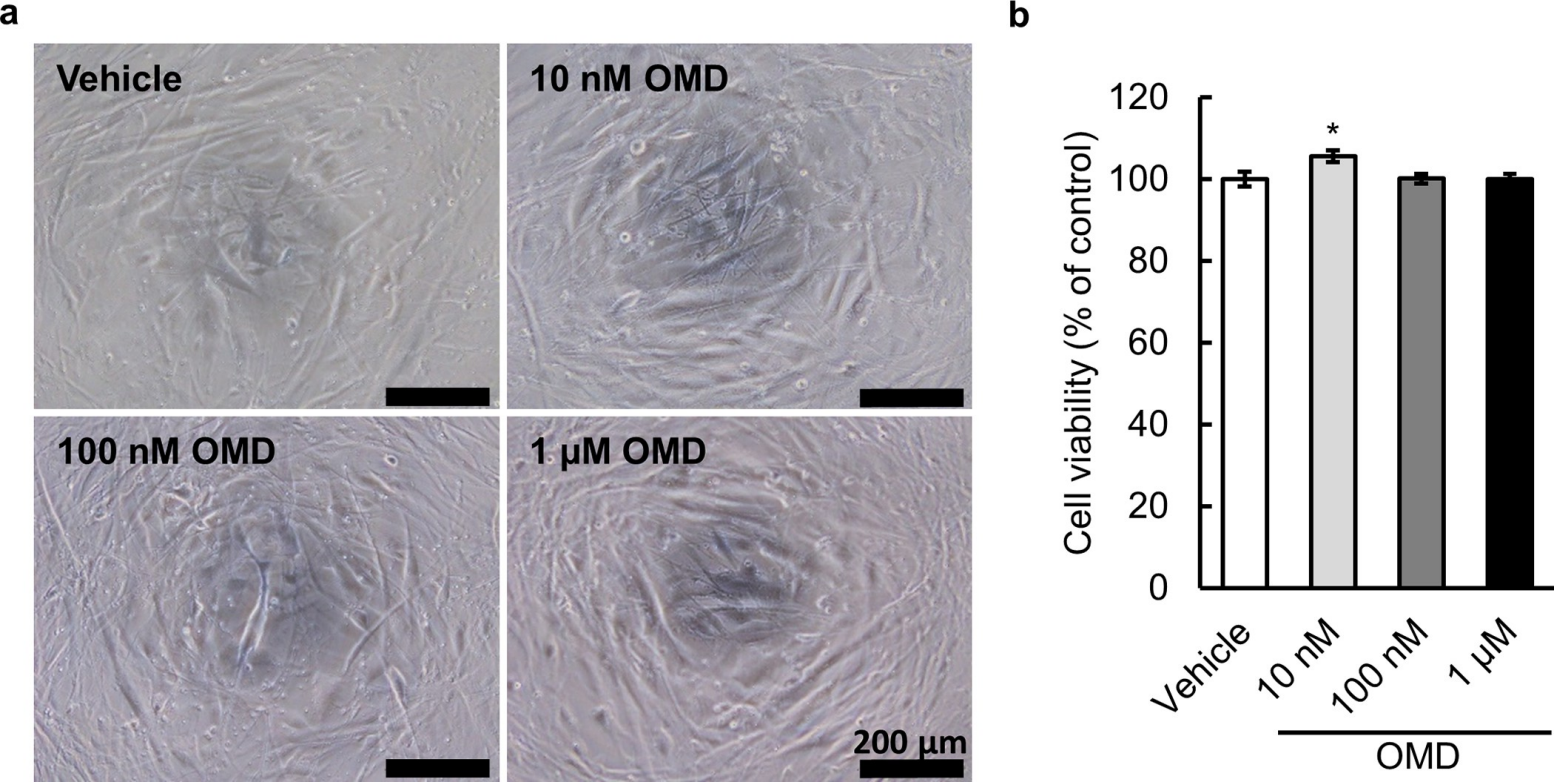
Effect of omidenepag (OMD) on human trabecular meshwork (HTM) cell viability. HTM cells were treated with vehicle or OMD (10 nM, 100 nM, and 1 μM) for 24 h, and cell viability was assessed using MTS assays. (**a**) Typical phase-contrast microscopic appearance of HTM cells after treatment. Scale bars = 200 μm. (**b**) Absorbance in each well was normalized to that in the vehicle-treated wells and presented as percentages. Each value represents the mean ± S.E. (n = 6). *P < 0.05, compared with the vehicle-treated group by Dunnett’s multiple comparison test. MTS, 3-(4,5-dimethylthiazol-2-yl)-5-(3-carboxymethoxyphenyl)-2-(4-sulfophenyl)-2H-tetrazolium.

To determine whether OMD has cytotoxicity in HTM cells, we first examined the effect of OMD at concentrations of 10 nM, 100 nM, and 1 μM for 24 h on cell viability under 2D culture conditions. Fig 1A shows no changes in the appearance of HTM cells after 24 h of OMD treatment at all three concentrations. Fig 1B shows the lack of cytotoxicity in all OMD-treated groups and only a slight increase (105.6%) in cell viability in the 10 nM OMD-treated group.

### Effect of OMD on the mRNA expression of ECM in HTM cells

**Fig 2:**
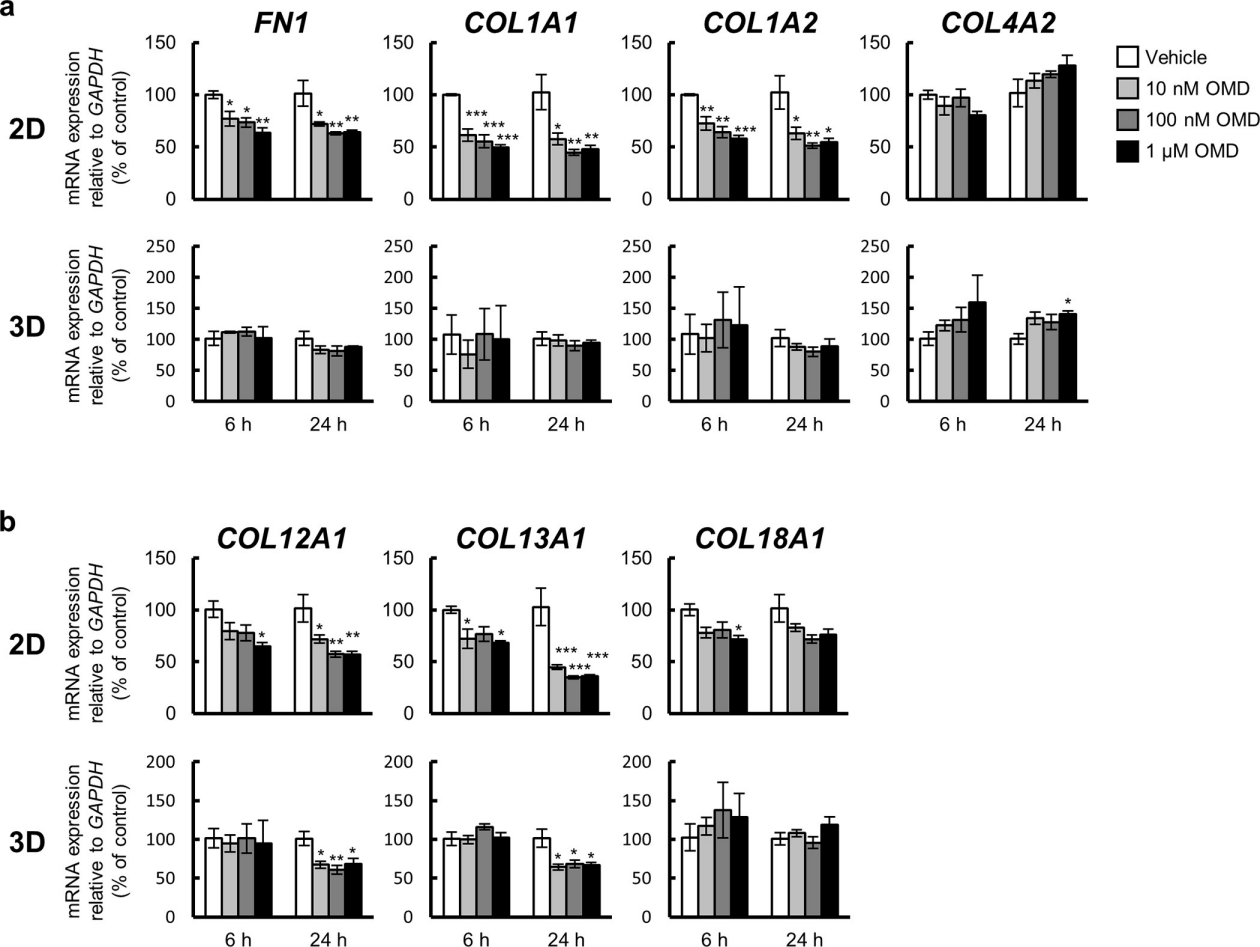
Effect of omidenepag (OMD) on extracellular matrix (ECM) gene expression in 2D- and 3D-cultured human trabecular meshwork (HTM) cells. 2D- or 3D-cultured HTM cells were treated with vehicle, 10 nM, 100 nM, or 1 μM OMD for 6 or 24 h, and the gene expression changes in ECMs [**(a)** *FN1*, *COL1A1*, *COL1A2*, and *COL4A2*, **(b)** *COL12A1*, *COL13A1*, and *COL18A1*] were assessed using quantitative real-time PCR. The mRNA expression level in each gene was normalized to that for *GAPDH* and presented as percentages relative to the vehicle-treated group. Each value represents the mean ± S.E. (n = 3). *P < 0.05, **P < 0.01, ***P < 0.001 compared with the vehicle-treated group by Dunnett’s multiple comparison test.

To investigate the effects of OMD on the mRNA expression of the ECM in HTM cells, the mRNA expression of fibronectin (*FN1*), collagen type I alpha 1 chain (*COL1A1*), collagen type I alpha 2 chain (*COL1A2*), collagen type IV alpha 2 chain (*COL4A2*), collagen type XII alpha 1 chain (*COL12A1*), collagen type XIII alpha 1 chain (*COL13A1*), and collagen type XVIII alpha 1 chain (*COL18A1*) was quantified using quantitative real-time PCR (Fig 2).

Under 2D culture conditions, the mRNA expression of *FN1*, *COL1A1*, *COL1A2*, *COL12A1*, and *COL13A1* was significantly downregulated in a concentration-dependent manner after 6 or 24 h of OMD treatment at all three concentrations. The results of all statistical analyses are shown in Table 2. *COL18A1* mRNA was significantly downregulated only after 6 h of treatment with 1 μM OMD. No significant changes in *COL4A2* mRNA expression were observed at any concentration or treatment period.

**Table 2:** P values for the gene expression changes in OMD-treated groups compared with the vehicle-treated group.

Gene	Culture condition	Treatment period (h)	Dunnett’s test P value in OMD-treated group		
			10 nM	100 nM	1 μM
*FN1*	2D	6	0.0350*	0.0168*	0.0029**
		24	0.0135*	0.0019**	0.0025**
	3D	6	0.8557 (N.S.)	0.8229 (N.S.)	0.9997 (N.S.)
		24	0.2694 (N.S.)	0.2170 (N.S.)	0.5082 (N.S.)
*COL1A1*	2D	6	0.0007***	0.0003***	0.0001***
		24	0.0205*	0.0052**	0.0072**
	3D	6	0.8872 (N.S.)	1.0000 (N.S.)	0.9977 (N.S.)
		24	0.9896 (N.S.)	0.6746 (N.S.)	0.9056 (N.S.)
*COL1A2*	2D	6	0.0054**	0.0010**	0.0004***
		24	0.0316*	0.0083**	0.0116*
	3D	6	0.9992 (N.S.)	0.9615 (N.S.)	0.9902 (N.S.)
		24	0.6468 (N.S.)	0.3428 (N.S.)	0.7094 (N.S.)
*COL4A2*	2D	6	0.5600 (N.S.)	0.9754 (N.S.)	0.1669 (N.S.)
		24	0.6666 (N.S.)	0.3883 (N.S.)	0.1520 (N.S.)
	3D	6	0.8780 (N.S.)	0.7320 (N.S.)	0.3010 (N.S.)
		24	0.0792 (N.S.)	0.1682 (N.S.)	0.0409*
*COL12A1*	2D	6	0.1504 (N.S.)	0.1237 (N.S.)	0.0184*
		24	0.0492*	0.0070**	0.0067**
	3D	6	0.9891 (N.S.)	1.0000 (N.S.)	0.9906 (N.S.)
		24	0.0214*	0.0086**	0.0240*
*COL13A1*	2D	6	0.0295*	0.0638 (N.S.)	0.0146*
		24	0.0006***	0.0001***	0.0001***
	3D	6	0.9994 (N.S.)	0.2782 (N.S.)	0.9951 (N.S.)
		24	0.0124*	0.0223*	0.0172*
*COL18A1*	2D	6	0.0641 (N.S.)	0.1014 (N.S.)	0.0193*
		24	0.2628 (N.S.)	0.0579 (N.S.)	0.1040 (N.S.)
	3D	6	0.9582 (N.S.)	0.6716 (N.S.)	0.8248 (N.S.)
		24	0.8479 (N.S.)	0.9488 (N.S.)	0.3170 (N.S.)
*MMP1*	2D	6	0.1450 (N.S.)	0.1343 (N.S.)	0.0642 (N.S.)
		24	0.2284 (N.S.)	0.0311*	0.1921 (N.S.)
	3D	6	0.9975 (N.S.)	0.7227 (N.S.)	1.0000 (N.S.)
		24	0.0193*	0.0339*	0.0308*
*MMP2*	2D	6	0.1509 (N.S.)	0.2783 (N.S.)	0.0288*
		24	0.3088 (N.S.)	0.0735 (N.S.)	0.1498 (N.S.)
	3D	6	0.9989 (N.S.)	0.9810 (N.S.)	0.8508 (N.S.)
		24	0.6104 (N.S.)	0.2856 (N.S.)	0.0700 (N.S.)
*MMP3*	2D	6	0.0766 (N.S.)	0.0471*	0.0330*
		24	0.0697 (N.S.)	0.0183*	0.0601 (N.S.)
	3D	6	1.0000 (N.S.)	0.9980 (N.S.)	0.6673 (N.S.)
		24	0.9235 (N.S.)	0.1949 (N.S.)	0.1814 (N.S.)
*MMP9*	2D	6	0.2686 (N.S.)	0.1110 (N.S.)	0.0680 (N.S.)
		24	0.5568 (N.S.)	0.4209 (N.S.)	0.8833 (N.S.)
	3D	6	0.8561 (N.S.)	0.9977 (N.S.)	0.4209 (N.S.)
		24	0.9937 (N.S.)	0.8838 (N.S.)	0.9939 (N.S.)
*MMP11*	2D	6	0.8584 (N.S.)	0.9900 (N.S.)	0.8662 (N.S.)
		24	0.8241 (N.S.)	0.9959 (N.S.)	0.7558 (N.S.)
	3D	6	0.0836 (N.S.)	0.1372 (N.S.)	0.8257 (N.S.)
		24	0.7044 (N.S.)	0.9992 (N.S.)	0.8420 (N.S.)
*MMP12*	2D	6	0.6537 (N.S.)	0.3020 (N.S.)	0.9571 (N.S.)
		24	0.0035**	0.0033**	0.0013**
	3D	6	0.9920 (N.S.)	0.9936 (N.S.)	0.6872 (N.S.)
		24	0.0918 (N.S.)	0.4956 (N.S.)	0.3962 (N.S.)
*MMP14*	2D	6	0.0001***	0.0001***	0.0000***
		24	0.1452 (N.S.)	0.0080**	0.0034**
	3D	6	0.9998 (N.S.)	0.9890 (N.S.)	0.6704 (N.S.)
		24	0.5992 (N.S.)	0.5110 (N.S.)	0.5357 (N.S.)
*MMP15*	2D	6	0.0289*	0.0156*	0.0053**
		24	0.0219*	0.0061**	0.0175*
	3D	6	0.9998 (N.S.)	0.8476 (N.S.)	0.2063 (N.S.)
		24	0.4737 (N.S.)	0.3173 (N.S.)	0.4516 (N.S.)
*MMP16*	2D	6	0.4794 (N.S.)	0.2708 (N.S.)	0.8713 (N.S.)
		24	0.0047**	0.0078**	0.0065**
	3D	6	0.9995 (N.S.)	0.9952 (N.S.)	0.9789 (N.S.)
		24	0.6542 (N.S.)	0.8057 (N.S.)	0.4501 (N.S.)
*MMP17*	2D	6	0.8188 (N.S.)	0.6390 (N.S.)	0.9981 (N.S.)
		24	0.7874 (N.S.)	0.4794 (N.S.)	0.4117 (N.S.)
	3D	6	0.8780 (N.S.)	0.8746 (N.S.)	0.3919 (N.S.)
		24	1.0000 (N.S.)	0.4254 (N.S.)	0.9999 (N.S.)
*MMP24*	2D	6	0.2069 (N.S.)	0.1283 (N.S.)	0.4015 (N.S.)
		24	0.0116*	0.0951 (N.S.)	0.0115*
	3D	6	0.9031 (N.S.)	0.2655 (N.S.)	0.9955 (N.S.)
		24	0.9036 (N.S.)	0.8347 (N.S.)	0.3973 (N.S.)
*TIMP1*	2D	6	0.0064**	0.0107*	0.0027**
		24	0.2134 (N.S.)	0.0877 (N.S.)	0.1495 (N.S.)
	3D	6	0.9998 (N.S.)	0.5857 (N.S.)	0.7965 (N.S.)
		24	0.9682 (N.S.)	0.3386 (N.S.)	0.5145 (N.S.)
*TIMP2*	2D	6	0.0230*	0.0205*	0.0094**
		24	0.0313*	0.0123*	0.0253*
	3D	6	0.9753 (N.S.)	0.7357 (N.S.)	0.6035 (N.S.)
		24	0.9619 (N.S.)	0.5219 (N.S.)	0.7236 (N.S.)
*TIMP3*	2D	6	0.5754 (N.S.)	0.7857 (N.S.)	0.9570 (N.S.)
		24	0.0481*	0.0021**	0.0003***
	3D	6	0.1230 (N.S.)	0.0529 (N.S.)	0.0180*
		24	0.0026**	0.0001***	0.0002***
*TIMP4*	2D	6	0.0695 (N.S.)	0.0350*	0.0086**
		24	0.0099**	0.0017**	0.0033**
	3D	6	0.9997 (N.S.)	0.7520 (N.S.)	0.9051 (N.S.)
		24	0.9963 (N.S.)	0.5939 (N.S.)	0.8105 (N.S.)

For each gene, the results of Dunnett’s multiple comparison test are presented. n = 3 for each sample; P values indicate comparisons with the vehicle-treated group.

Under 3D culture conditions, *COL12A1* and *COL13A1* mRNA expression was significantly downregulated after 24 h treatment with OMD at all three concentrations, and *COL4A2* mRNA expression was significantly upregulated only when treated for 24 h with 1 μM OMD.

The mRNA expression of *FN1*, *COL1A1*, *COL1A2*, and *COL18A1* did not show significant changes at any concentration or treatment periods.

In the 2D and 3D culture experiments, the shared gene expression changes were the downregulation of two ECM genes, *COL12A1* and *COL13A1*.

### Effect of OMD on the mRNA expression of MMPs and TIMPs in HTM cells

**Fig 3:**
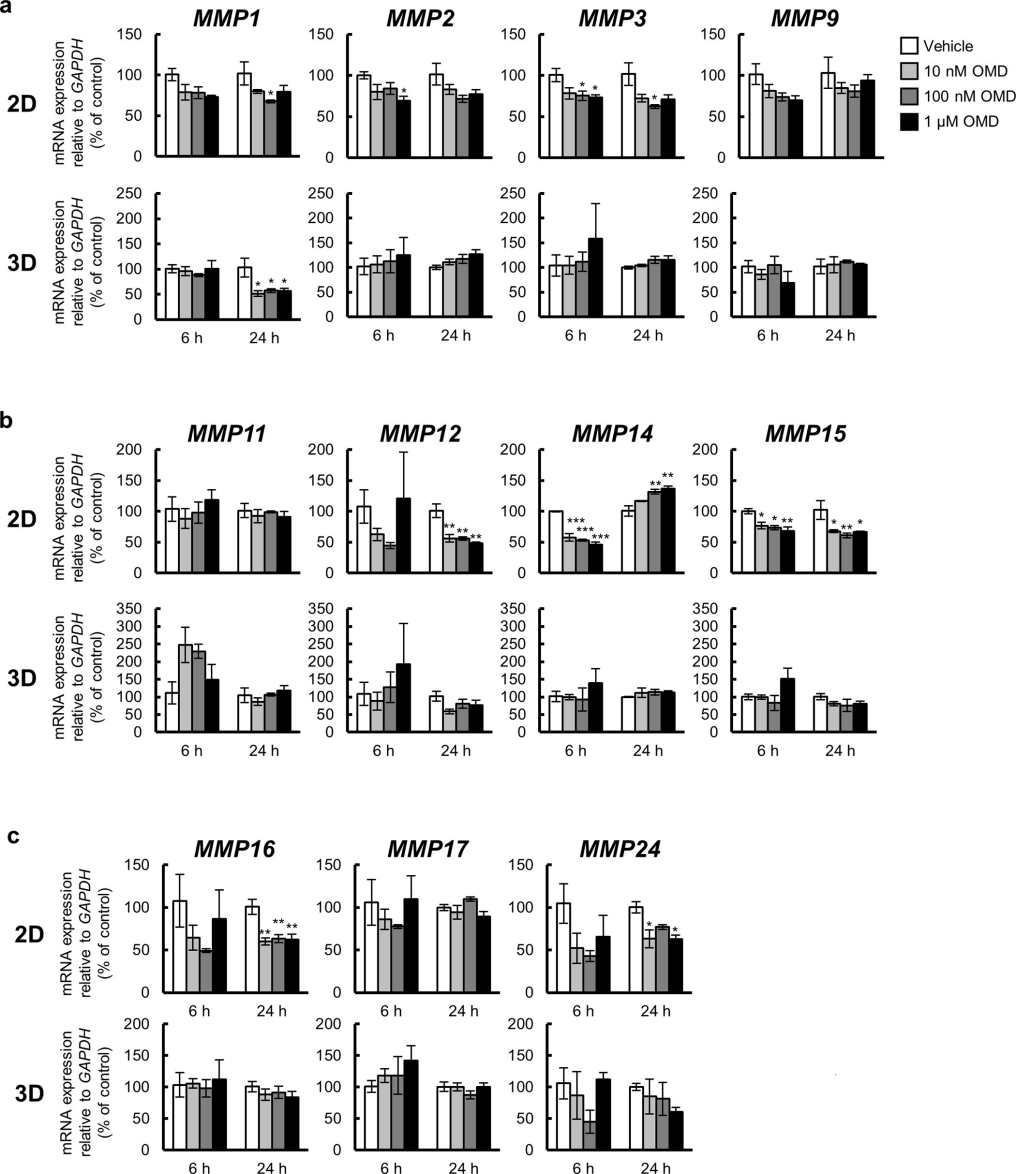
Effect of omidenepag (OMD) on matrix metalloproteinase (MMP) gene expression in 2D- and 3D-cultured human trabecular meshwork (HTM) cells. 2D- or 3D-cultured HTM cells were treated with vehicle, 10 nM, 100 nM, or 1 μM OMD for 6 or 24 h, and the gene expression changes of MMPs [**(a)** MMP1, MMP2, MMP3, and MMP9, **(b)** MMP11, MMP12, MMP14, and MMP15, **(c)** MMP16, MMP17, and MMP24] were assessed using quantitative real-time PCR; gene expression was assessed using real-time PCR. The mRNA expression level in each gene was normalized to that for *GAPDH* and presented as percentages relative to the vehicle-treated group. Each value represents the mean ± S.E. (n = 3). *P < 0.05, **P < 0.01, ***P < 0.001 compared with the vehicle-treated group by Dunnett’s multiple comparison test.

**Fig 4:**
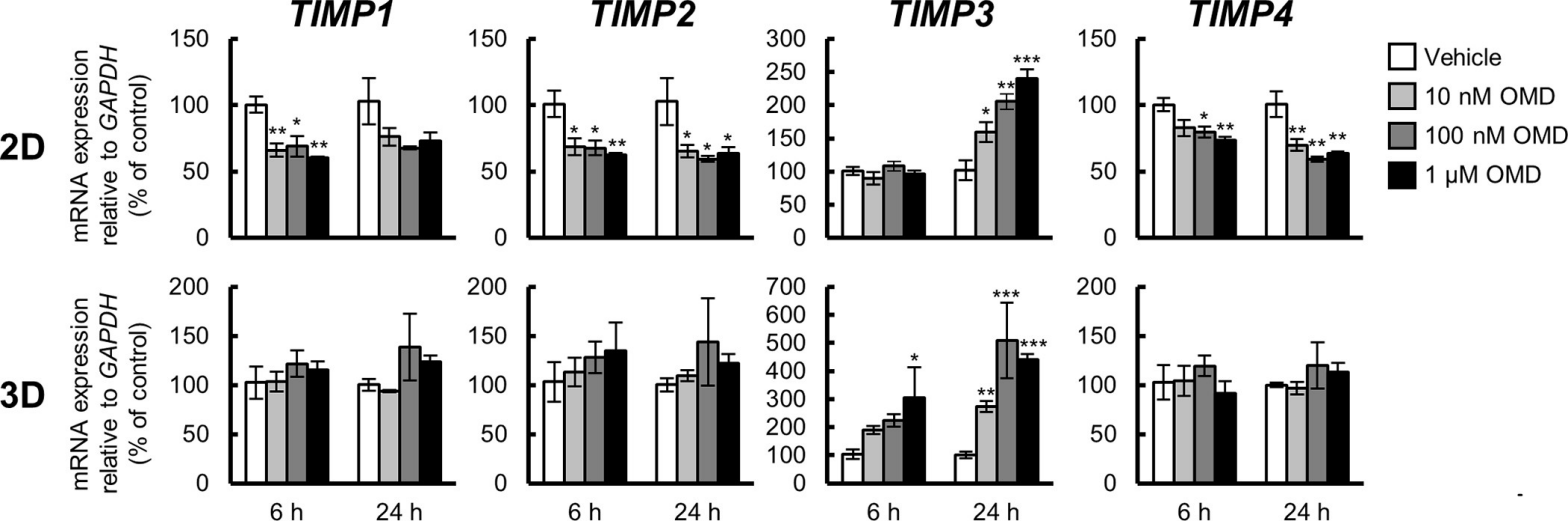
Effect of omidenepag (OMD) on tissue inhibitors of metalloproteinase (TIMP) gene expression in 2D- and 3D-cultured human trabecular meshwork (HTM) cells. 2D- or 3D-cultured HTM cells were treated with vehicle, 10 nM, 100 nM, or 1 μM OMD for 6 or 24 h, and the gene expression changes of TIMPs (*TIMP1*, *TIMP2*, *TIMP3*, and *TIMP4*) were assessed using quantitative real-time PCR. The mRNA expression level in each gene was normalized to that of *GAPDH* and presented as percentages relative to the vehicle-treated group. Each value represents the mean ± S.E. (n = 3). *P < 0.05, **P < 0.01, ***P < 0.001 compared with the vehicle-treated group by Dunnett’s multiple comparison test.

To determine the effect of OMD on the mRNA expression of *MMPs* and *TIMPs* in HTM cells, that of *MMP1*, *MMP2*, *MMP3*, *MMP9*, *MMP11*, *MMP12*, *MMP14*, *MMP15*, *MMP16*, *MMP17*, and *MMP24*, and *TIMP1*, *TIMP2*, *TIMP3*, and *TIMP4* was quantified using quantitative real-time PCR (Figs 3 and 4).

Under 2D culture conditions, the mRNA expression of *MMP2* was significantly downregulated after 6 h, and that of *MMP1*, *MMP12*, *MMP16*, and *MMP24* was significantly downregulated after 24 h treatment with OMD (Fig 3). The mRNA expression of *MMP3* and *MMP15* was significantly downregulated after both 6 and 24 h of OMD treatment. *MMP14* mRNA expression was significantly downregulated after 6 h and upregulated after 24 h of OMD treatment. *TIMP1* mRNA expression was significantly downregulated following 6 h of OMD treatment and although it continued a downregulation trend, it was not significant 24 h post treatment (Fig 4). The mRNA expression of *TIMP2* and *TIMP4* was significantly downregulated after both 6 and 24 h of OMD treatment. *TIMP3* mRNA expression was significantly upregulated 24 h after treatment.

Under 3D culture conditions, *MMP1* mRNA expression was significantly downregulated 24 h after treatment, while *MMP11* appeared to be upregulated 6 h after treatment (Fig 3). No significant changes in mRNA expression of *TIMP1*, *TIMP2*, and *TIMP4* were observed at any concentration or treatment period (Fig 4). *TIMP3* mRNA expression was significantly upregulated after 6 and 24 h of treatment.

In the 2D and 3D culture experiments, the shared gene expression changes were the downregulation of *MMP1* (Fig 3) and upregulation of *TIMP3* (Fig 4).

## Discussion

Owing to the limitations of 2D culture in evaluating drug efficacy, i.e., occasional inaccurate representation of tissue cells *in vitro* and different behaviors compared with those *in vivo*, 3D culture is being applied as an alternative to bridge the gap between 2D culture and animal models [38–40]. These are unique systems that can reflect cell–cell and cell–ECM interactions *in vivo* [41,42] and maintain their function and structure [43,44]. As for the TM tissue, it shows cell–ECM interactions and is involved in the regulation of aqueous humor dynamics because of its unique biomechanical properties [45,46]. Fibronectin is abundant in the TM tissue [47] where multiple subtypes of collagen (I, IV, XII, XVIII) are expressed [48–50]. MMP1, 2, 3, 9, 11, 12, 14, 15, 16, 17, and 24 are involved in the degradation of the ECM, while TIMPs suppress MMP activity by forming complexes with MMPs [51,52]. Considering these advantages, we used a 3D culture system that does not require specific scaffolds and is capable of constructing 3D tissue with uniform thickness, unlike spheroids [35], to evaluate the effects of OMD in the TM tissue *in vitro*.

In the present study, we evaluated the effects of OMD on the mRNA expression of the ECM, MMPs, and TIMPs in 2D- and 3D-cultured HTM cells, which have been confirmed to be TM cells that increase myocilin gene expression in response to steroids [31,32], in order to elucidate the IOP-lowering mechanism of OMDI that promotes the conventional outflow pathway.

According to the monkey pharmacokinetics data used for the new drug application of OMDI ophthalmic solution 0.002%, the OMD concentration in the TM tissue after instillation was estimated at 59.8 nM. Considering the agonistic activity of OMD to EP2 receptor (EC_50_  =  8.3 nM), although a concentration range of 10–100 nM may be sufficient for evaluation, we extended the range from 10 nM to 1 μM in the present study. A significant increase in HTM cell viability was observed in the 10 nM OMD-treated group. However, the difference between the increase in cell viability in the vehicle-treated group and 10 nM OMD-treated group was only 5.6%, and no increase in HTM cell viability was observed at higher concentration rages (100 nM and 1 μM). Therefore, the increase in HTM cell viability in the 10 nM OMD-treated group was considered to be incidental and unrelated to cell proliferation.

Although 1 μM is higher than the estimated TM tissue concentrations in clinical practice, some genes with significant expression changes showed a concentration-dependent response within the concentration range of 10 nM to 1 μM (*FN1*, *COL1A1*, *COL1A2*, *COL12A1*, and *COL13A1*) in 2D culture. Conversely, among the genes that showed significant expression change in 3D culture, only *COL4A2* showed a significant expression change at 1 μM, and the other genes (*COL12A1*, *COL13A1*, *MMP1*, and *TIMP3*) showed significant expression changes from 10 nM to 1 μM. In clinical practice, the TM tissue is expected to be exposed to OMD at concentrations of approximately 50 nM. Further expression changes were observed at higher concentrations in 2D culture, while almost all responses to OMD in 3D culture reached a plateau at 10 nM, which may appropriately reflect the *in vivo* response to OMD after instillation.

These differences in expression between the 2D and 3D cultures may be attributed to changes in cellular response due to differences in culture environments. Drug sensitivity is increased in 2D culture systems [43], and hence the mRNA expression under the 2D culture condition may be overestimated. In addition, we have unpublished background data that indicate that the mRNA expression level of EP2 receptor in 2D-cultured HTM was higher than that of in 3D-cultured HTM; therefore, the increased drug sensitivity in 2D-cultured HTM was thought to be a plausible event. However, because such increased drug sensitivity under 2D culture conditions is useful for screening to broadly capture gene expression changes, we first evaluated the effect of OMD on HTM cells in 2D culture. Considering this, many changes in mRNA expression were observed as described above. In contrast, the mRNA expression of only a few genes, such as *COL12A1* and *COL13A1*, was significantly downregulated under 3D culture conditions. Although we have not assessed the effect of OMD on collagen type XII and XIII proteins yet, the cellular responses to OMD in 3D culture conditions are expected to be similar to those *in vivo* because HTM cells under 3D culture conditions retain more cellular characteristics *in vivo* compared to 2D cultures.

Among the above gene expression changes, we hypothesized that downregulation of the ECM-related genes, especially *COL12A1* and *COL13A1*, might have caused the increase in aqueous humor outflow via the conventional outflow pathway followed by the decrease in IOP.

COL12 is a type of non-fibrillar collagen called fibril-associated collagens with interrupted triple helices (FACIT) that can form bonds between fibrils [49,53]. *COL12* expression increases in response to mechanical stimuli [54–57], while TM cells sense elevated IOP as a mechanical stretching [49]; hence, there is a possibility that COL12 expression and IOP might be correlated. Further, an EP2 receptor agonist (AH13205) has been found to contribute to the relaxation of isolated TM tissue [58]. Under the 3D culture conditions used in the present study, in addition to the direct downregulation by EP2 stimulation, the physical stimulus of TM relaxation by EP2 stimulation may also result in a significant downregulation of *COL12A1* expression. Based on these findings, it is possible that the IOP-lowering effect of OMDI is attributed to the reduction in aqueous humor outflow resistance caused by the physical relaxation of the TM tissue and reductions in mechanical stretching and *COL12* expression in TM tissue, which may contribute to ECM remodeling and consequently IOP reduction.

COL13 is a transmembrane collagen [59]. Its localization and function in the TM tissue and association with glaucoma are still unknown. However, *COL13* has cell adhesion-related functions at various cell–ECM junctions [60]. As the TM tissue is composed of TM cells and ECM, there is a possibility that *COL13* may be involved in cell–cell and cell–ECM adhesion of TM cells and the regulation of aqueous humor outflow resistance. Under this assumption, OMD downregulates *COL13A1* expression and consequently contributes to the reduction of aqueous humor outflow resistance in the TM tissue.

In the present study, *COL12A1* and *COL13A1* were downregulated following OMD treatment in 2D and 3D cultures. It is well known that reduced amount of ECM contributes to decreased outflow resistance [25,61–63]. Taken together, downregulation of those two genes may be one of the factors responsible for the IOP-lowering effect of OMDI.

However, there are certain limitations to this study. As the results in the present study were obtained from only one lot of HTM cells isolated from a single donor, they may be specific to this primary HTM cell alone; hence, further study might be necessary to evaluate whether the same results will be obtained using other HTM cells from other donors. Furthermore, we only evaluated gene expression using cell culture. Further studies to determine the effects of OMD on ECM protein expression and MMP activities under 3D culture conditions are currently underway. 3D-cultured tissue may reflect the TM tissue and the microenvironment of TM *in vivo* more accurately than 2D-cultured cells do. However, because the 3D cultured tissue does not fully reflect the *in vivo* TM structure, it should be noted that the results in the current study may not fully reproduce the *in vivo* TM response. As the accumulation of ECM in the TM tissue followed by increased conventional outflow resistance is an important factor that elevates IOP in glaucoma [24–26], suppression of ECM accumulation in the TM tissue is important for long-term IOP control in glaucoma treatment. Nonetheless, as OMDI suppresses the expression of *COL12A1* and *COL13A1*, which are part of the ECM components, OMDI could maintain the conventional outflow before ECM accumulation progresses and the TM tissue becomes completely occluded. Further clarification of the mechanism of OMDI in the conventional outflow pathway may enhance its clinical use, such as administering OMDI when the conventional pathway is relatively healthier. In other words, OMDI may be feasibly administered when patients are still in earlier stages of glaucoma and have healthier TM tissue, thereby maintaining the conventional outflow. Such practices may enhance the use of OMDI among the many existing anti-glaucoma drugs in clinical practice.

## Supporting information

S1 Fig Effect of dexamethasone (DEX) on myocilin (MYOC) mRNA gene expression in HTM cells. HTM cells were seeded in 6-well plates pre-coated with poly-L-lysine at a density of 1.0 × 10^4^ cells/well (1.1 × 10^3^ cells/cm^2^) and incubated for 24 h. Subsequently, these cells were treated with vehicle (TMCM with 0.01% DMSO) or 500 nM DEX for 6 d, and the changes in the gene expression of MYOC were assessed using quantitative real-time PCR. The mRNA expression level was normalized to that for GAPDH and presented as percentages relative to the vehicle-treated group. Each value represents the mean ± S.E. (n = 6). *P < 0.05 compared with the vehicle-treated group by Student’s t-test. The primer set for MYOC was pre-designed and purchased from Takara Bio (Shiga, Japan; Cat. # HA205173).
(TIF)

S1 File PCR data for S1 Fig. (XLSX)

S2 File MTS assay data for Fig 1. (XLSX)

S3 File PCR data for Fig 2A and 2B (2D). (XLSX)

S4 File PCR data for Fig 2A and 2B (3D). (XLSX)

S5 File PCR data for Fig 3A–3C (2D). (XLSX)

S6 File PCR data for Fig 3A–3C (3D). (XLSX)

S7 File PCR data for Fig 4 (2D). (XLSX)

S8 File PCR data for Fig 4 (3D). (XLSX)
