# Supplementary figures and images for: Downregulation of COL12A1 and COL13A1 by a selective EP2 receptor agonist, omidenepag, in human trabecular meshwork cells
Source: PLoS One. 2023 Jan 11;18(1):e0280331. doi: 10.1371/journal.pone.0280331 (PMC9833537; doi:10.1371/journal.pone.0280331)

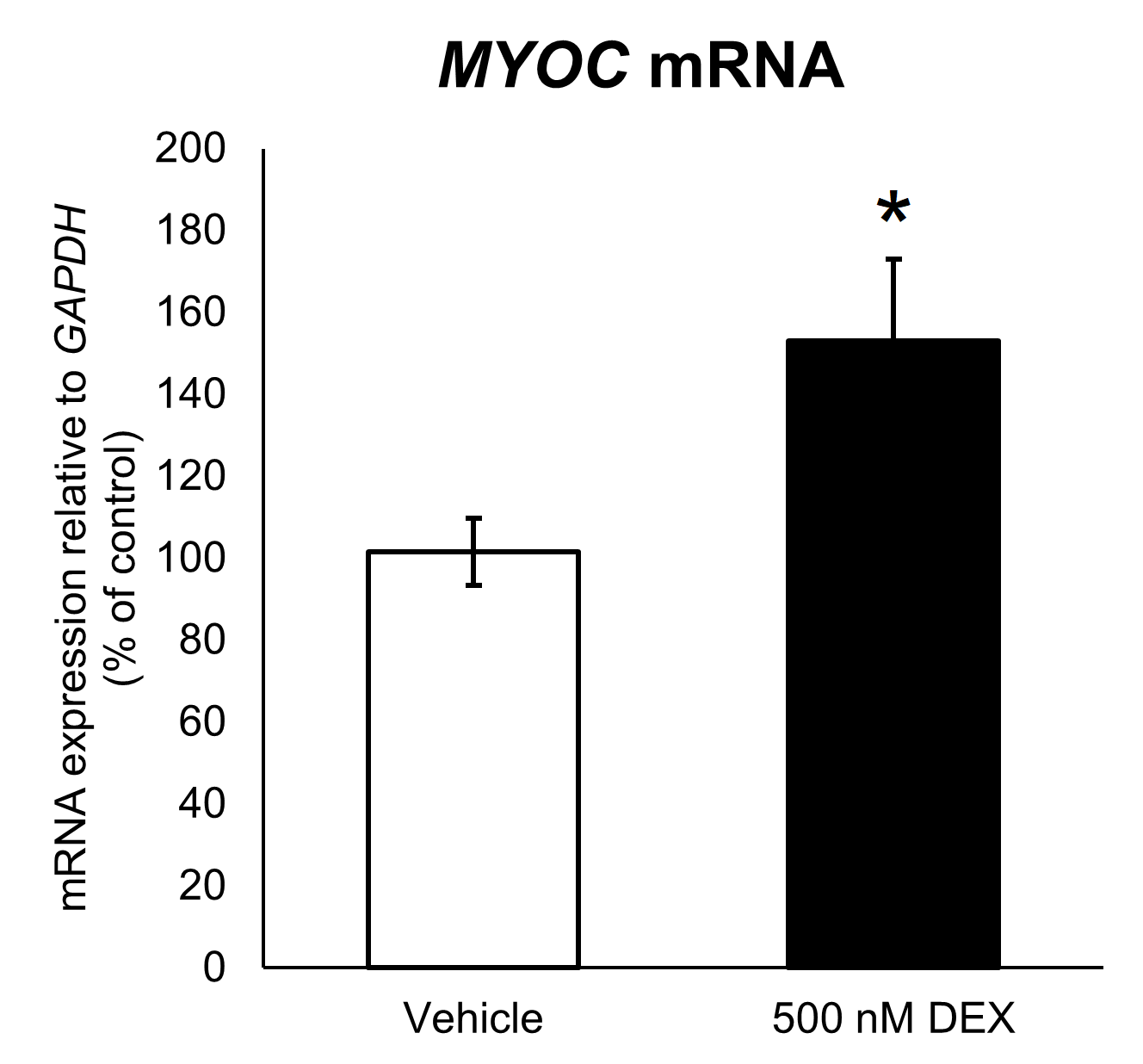

Supplement: S1 Fig — HTM cells were seeded in 6-well plates pre-coated with poly-L-lysine at a density of 1.0 × 10^4^ cells/well (1.1 × 10^3^ cells/cm2) and incubated for 24 h. Subsequently, these cells were treated with vehicle (TMCM with 0.01% DMSO) or 500 nM DEX for 6 d, and the changes in the gene expression of MYOC were assessed using quantitative real-time PCR. The mRNA expression level was normalized to that for GAPDH and presented as percentages relative to the vehicle-treated group. Each value represents the mean ± S.E. (n = 6). *P < 0.05 compared with the vehicle-treated group by Student’s t-test. The primer set for MYOC was pre-designed and purchased from Takara Bio (Shiga, Japan; Cat. # HA205173). (TIF) [file pone.0280331.s001.tif]
